# The role of artificial intelligence in cardiovascular research: Fear less and live bolder

**DOI:** 10.1111/eci.14364

**Published:** 2025-04-07

**Authors:** Alessandro Scuricini, Davide Ramoni, Luca Liberale, Fabrizio Montecucco, Federico Carbone

**Affiliations:** ^1^ Department of Internal Medicine University of Genoa Genoa Italy; ^2^ IRCCS Ospedale Policlinico San Martino, Genoa – Italian Cardiovascular Network Genoa Italy

**Keywords:** artificial intelligence, chatbots, ChatGPT, large language models, medical writing, natural language understanding

## Abstract

**Background:**

Artificial intelligence (AI) has captured the attention of everyone, including cardiovascular (CV) clinicians and scientists. Moving beyond philosophical debates, modern cardiology cannot overlook AI's growing influence but must actively explore its potential applications in clinical practice and research methodology.

**Methods and Results:**

AI offers exciting possibilities for advancing CV medicine by uncovering disease heterogeneity, integrating complex multimodal data, and enhancing treatment strategies. In this review, we discuss the innovative applications of AI in cardiac electrophysiology, imaging, angiography, biomarkers, and genomic data, as well as emerging tools like face recognition and speech analysis. Furthermore, we focus on the expanding role of machine learning (ML) in predicting CV risk and outcomes, outlining a roadmap for the implementation of AI in CV care delivery. While the future of AI holds great promise, technical limitations and ethical challenges remain significant barriers to its widespread clinical adoption.

**Conclusions:**

Addressing these issues through the development of high‐quality standards and involving key stakeholders will be essential for AI to transform cardiovascular care safely and effectively.

## Graphical abstract

The pathways for implementing artificial intelligence (AI) in cardiovascular care are complex and interconnected. Beyond advancing multimodal data analysis techniques (e.g. machine learning, deep learning, neural networks and natural language processing), it is crucial to address requirements such as data quality, ethics, equity and standardized best practices. Collaboration with regulatory authorities, AI‐focused journals and stakeholders is vital to ensure safety, fairness and external validation, ultimately fostering the ethical and effective integration of AI into clinical practice.

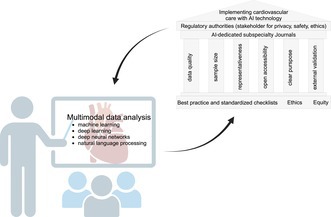

## 1 INTRODUCTION

The questions we ask remain the same: *what thinking means* and *whether machines can think*. Unfortunately, the popular debate surrounding artificial intelligence (AI) still bases itself on these *wrong* assumptions. As early as 1950, Alan Turing—a visionary pioneer of computer science—chose not to do so. As a brilliant logician, he believed those questions meaningless and replaced them with a more pragmatic question: ‘Can a computer communicate in a way indistinguishable from human?’.^1^ This approach is also known as the Turing test, or imitation game. While Turing rejected the claims that ‘a machine can think’ or ‘a machine cannot make mistakes’, new questions have arisen: ‘can AI learn, and if so, how?’. The later theory of language games by Wittgenstein offers additional valuable insights. He argued that the meaning of a word or language is shaped by how people engage in. Following this logic, AI begins to learn a language through exposure, and large language models (LLMs) like ChatGPT‐3 are trained on such a vast array of ‘language games’ that it would take thousands of years for a person to read through all of its training data. Setting aside cultural and philosophical speculation, Turing had been developing the concept of machine intelligence since at least 1941, but it took him 10 years of exploration before formally establishing the field of AI. From ELIZA (1966), a plethora of programs able to ‘play the imitation game’ has been developed. Yet, the crossover between AI and health science is relatively recent, notably exemplified by the rapid adoption of ChatGPT among healthcare workers, now approaching nearly 20 percent.^2^ Such a ‘data (r)evolution,’ with the ever‐increasing collection of data, has had a significant impact on medicine, particularly in the field of cardiology. From epidemiology to data science, new independent disciplines are emerging within cardiology and deserve to be summarized here, along with efforts to explore their current and future applications.

## 2 DATA TYPES AND PROCESSING IN CARDIOVASCULAR FIELD: AN EVER‐EXPANDING UNIVERSE

The beginning of modern cardiovascular (CV) research dates back from early 1950. At that time, the NIH's Framingham Heart Study represented one of the first long‐term cohort studies of its kind and was considered the *crown jewel* of epidemiology. Patricia McNemara stayed with the study for more than 30 years as the data manager and analyst who computed and stored millions of health measures. There were no rules to follow except the quote ‘learning by doing’. ‘You see, we were trying to look at eighty variables in 5209 people. We had no copy machines. We had to use carbon paper. No electronic calculators!’ She recalled. That was another era. ‘The IBM punch cards, they were the only technological advance that was there early on. This IBM card‐sorting machine was like a big piano. You'd put all these punch cards in, and it would take eight hours to do what you could do in half a second now on a computer. Not only that, it would mangle a dozen or so cards every now and then. You'd have to stop everything and repunch them’.^3^ Despite these challenges, the study's rigorous design and statistical methods have stood the test of time, producing statistically reliable findings that have held up for half a century.

Contemporary cardiology has witnessed a whirlwind of progress, much of it stemming from the cumulative insights gained through multimodal data.^4^ Available data now ranges from structured to semi‐structured and unstructured data, each varying in terms of collection, storage and susceptibility to specific errors during AI analysis (summarized in Table 1). The computational simplicity of AI in developing machine learning (ML) algorithms has fueled early studies focused on leveraging structured tabular data. While ML indeed enables the capture of non‐linear causal relationships, which are expected to perform better on real‐world data, this assumption has recently been challenged.

**TABLE 1:** Glossary for data categorization in cardiovascular field.

Data type	Clinical	Instrumental	Characteristics
Structured data	Patient demographics recorded in a standardized EMR system Laboratory results	ECG readings	Highly organized and formatted Easily searchable and analysable Often stored in databases Allows for statistical analysis, predictive modelling Enable comparisons across patients or populations
Semi‐structured data	Clinical notes in EMR: mostly unstructured text, but with some tagging or categorization (e.g. symptoms, diagnoses) Data from wearable device (e.g. activity tags, metadata)	Echocardiography data (e.g. metadata like patient ID, date of scan and machine settings)	Lacks strict formatting but retains some organizational properties (e.g. metadata) More flexible but requires some processing for analysis
Unstructured data	Genomics data: raw unstructured gene sequencing	Raw data from echocardiograms, MRIs, or CT scans	Lacks any predefined organizational format More complex to analyse, often involving large volumes of text, images, or other data requiring advanced techniques for processing

Abbreviations: CT, computerized tomography; ECG, electrocardiography; EMR, electronic medical record; MRIs, magnetic resonance imaging.

Instead, unstructured data is arguably the most fascinating mine of information, now accessible through AI but otherwise hidden to humans. The progress in deep learning models (e.g. recurrent/convolutional neural networks, transformers, attention mechanisms, bidirectional models, encoder‐decoder architecture and transfer learning with pre‐trained models) enables the processing of different raw unstructured biometric signals and image. When applied to natural language processing (NLP), deep learning allows for the analysis of a large volume of patient information from electronic health records (EHR, e.g. medical history, results of examinations and management) with diagnostic/prognostic purposes. NLP already outperform clinicians in diagnosing heart failure (HF) with preserved ejection fraction, leading to improved prediction of hospitalization and mortality.^5^ However, its processing complexity oscillates between the need of pre‐processing to reduce background noise and the advantage in retaining some variability and data heterogeneity to maintain the AI model applicable in a meaningful clinical context.^6^

## 3 HOW ADVANCES IN ARTIFICIAL INTELLIGENCE ARE TRANSFORMING CLINICAL PRACTICE IN CARDIOLOGY

The heterogeneity and polygenic background of CV disease require substantial effort to integrate multimodal data, from addressing its complexity to tailoring personalized treatment. Cardiac electrophysiology, imaging, angiography, biomarker and genomic data, as well as emerging areas like facial recognition and speech analysis, are subspecialties where the application of AI is rapidly advancing.

### 3.1 Cardiac electrophysiology

An accurate interpretation of the electrocardiogram (ECG) is a critical yet time‐consuming task for skilled clinicians, as the interpretation is often not straightforward. AI has emerged as a valuable tool in electrophysiology to address these demands. Deep learning algorithms are indeed increasingly being used to interpret EGC with greater accuracy than clinicians.^7^, ^8^ Beyond their expected contribution for diagnosis in emergency settings, AI‐enabled ECG algorithms can also identify patients with paroxysmal atrial fibrillation during sinus rhythm.^9^ Consequently, the need to incorporate AI into portable devices (e.g. portable electrocardiographs and smartwatches) has been emphasized even in the recently updated ESC guidelines.^10^ Despite confirmatory evidence from a recent meta‐analysis on 26 studies involving 113,784 patients, the real impact on health outcomes remains limited and faces ethical concern, mainly related to the risk of over‐ or under‐prescription of anticoagulant therapies.^11^

### 3.2 Cardiac imaging

AI is upfront in cardiac imaging and represents a paradigm shift for its application in CV medicine. ML is a critical component for sophisticate image analysis using robotics and computer vision, enabling the acquisition and processing vast amounts of data. The deployment of artificial neural networks in deep learning models enhance—with a continuous learning process—data analysis and decision making.^12^, ^13^ These applications now extend from echocardiography^14^ to computed tomography (CT),^15^ cardiac magnetic resonance (CMR),^16^, ^17^, ^18^, ^19^ and positron emission tomography (PET).

A truly innovative advancement in this field is the use of deep learning analyses of dynamic coronary fluid based on fractional flow reserve (FFR) in tomography angiography, which now candidates as the next tool for implementing image interpretation. Such a tool can also facilitate more tailored lipid‐lowering therapies by accurately monitoring coronary plaque regression.^20^ Similarly, preliminary data support a role of AI in improving the assessment of myocardial perfusion and flow reserve through PET.^21^

### 3.3 Interventional cardiology

The growing amount of data generated by interventional cardiology is paving the way for more advanced and highly sophisticated AI applications. This technology enables more in‐depth analyses of intracoronary images, particularly when applied to optical coherence tomography in cases of complex atherosclerotic lesions.^22^ By providing real‐time estimations of the extent and severity of atherosclerotic disease, AI enhances the operator's decision‐making process.^23^ AI‐assisted analysis of images may help resolve the ongoing controversy surrounding the use of FFR, a recommended but still time‐consuming and technically challenging procedure. In this specific area, AI algorithms are shortening the procedure while maintaining excellent accuracy.^24^

AI offers substantial benefits in structural cardiology, particularly using virtual reality in three‐dimensional heart models. This technology allows for the simulation of valve repair procedures, enabling more accurate planning. The detailed analysis of anatomical models, combined with a wide range of demographic, biochemical and clinical data, assists clinicians in navigating the complex clinical workflow. It also helps patients provide truly informed consent to the procedure.^25^

### 3.4 Biomarker data and genomics

Biomarkers are the cornerstone for the multimodal data application of AI. They represent a wealth of information in CV medicine and promise advance in diagnosis, monitoring and treatment strategies. For over a decade, the use of ML‐based algorithms has been used to leverage individualized patient‐specific data and the associated metabolic/genomic profile to improve CV risk assessment. Despite routinely tested for improving CV stratification, they didn't yet get potential for clinical translation.^26^, ^27^, ^28^, ^29^, ^30^, ^31^, ^32^, ^33^ Since these models are usually built on retrospective cohorts without external validation, they are at risk of overfitting bias and a ‘black box’ prediction.

While delving deeper into ML algorithms, biomarkers are instead increasingly easier to integrate multimodal data from traditional diagnostic workflows, as shown in tracking a video‐based—specifically CMR—progression of aortic valve stenosis^34^ and the prediction of major cardiac events outperforming CT scans for CAC score, number of coronary lesions, aortic valve calcium score, EAT volume and attenuation.^35^

Beyond a mere predictive outlook, AI is also uncovering cardiogenomics in ways that were previously unattainable. An in silico score for coronary artery disease (CAD) was finally built and capable of capturing its underdiagnosis, severity and progression.^36^ The potential of AI‐driven genomic analysis is growing rapidly, but much remains unexplored. Besides attempts at integrating traditional workflow^37^, ^38^ and CV risk stratification,^39^ the greatest expectations are related to the potential of AI in accelerating the discovery of targets for drug^40^ and promoting tailored treatment strategies.

### 3.5 Face recognition and speech analysis

Facial recognition is used daily to enable secure unlocking of our smartphones, while speech analysis powers AI‐driven customer service chatbots that respond to our queries through NLP. Quite surprisingly, face recognition and speech analysis are now emerging as innovative AI tools with exciting potential for CV medicine and beyond. AI algorithms may use face characters to provide insights on subject's health. Wrinkles and earlobe creases have been linked with CAD in a very low‐cost and harmless fashion.^41^, ^42^ Similarly, AI may extrapolate information on individual's health from vocal characteristics. A recently developed mobile app now predicts hospitalizations for HF with an over 75% accuracy, up to 3 weeks in advance, by analysing changes in speech.^43^, ^44^ By leveraging the autonomic nervous system's influence on vocal characteristics and CV function, this approach could offer insights into a patient's heart health in a user‐friendly way.^45^

## 4 BACK TO THE FUTURE: AI‐ASSISTED CLINICAL DECISION PROCESS AND OUTCOME PREDICTION

Being a prototype of multimodal data, clustering observation, clinical examination, laboratory and instrumental finding, diagnostic algorithms are currently upfront for testing the predictive potential of AI. In the CV field, they are widely used in emergency and short‐term settings, exactly where ML and NLP may compete with conventional risk models. The diagnostic performance of the ML algorithm CoDe‐ACS consistently overcomes the traditional diagnosis of ACS by troponin assay. Such a predictive performance is independent of the timing of serial measurement and extends towards the risk of cardiac death at 30 days.^46^, ^47^

When considering in‐hospital and long‐term mortality, the potential of ML becomes increasingly evident. Applying ML models to established clinical scores offers a data‐driven approach accounting for time‐dependent and non‐linear variable interactions. Notably, the GRACE (Global Registry of Acute Coronary Events) 2.0 score has been ‘dissected’ through AI, revealing sex‐specific performance differences. After external validation, the GRACE 3.0 score led to a significant female reclassification towards the high‐risk group.^48^ Since then, the application of ML to CV risk and outcome prediction has exploded, and an ever‐growing amount of study seems destinated to reshape CV prediction.^49^, ^50^, ^51^

## 5 WILL AI IMPLEMENT CARDIOVASCULAR CARE DELIVERY? OUTLOOK AND CHALLENGES

While contemporary application of AI focuses on building the best tool optimizing the performance in standard domains, the future is on the horizon. As more as deep learning models leverage big data, they move from a general standardization to being fine‐tuned tools for local populations and minorities.^52^ Their ability to continuously leverage them during deployment indeed enhance longitudinal performance and may help in addressing population drifts.^53^ Moreover, patient population may change over time, and as AI models constantly learn, they adapt and stay up‐to‐date, critically maintaining their validity.^48^, ^49^, ^50^, ^54^ Ultimately, accelerating and standardizing time‐consuming tasks not only maximizes patient care but it is intended to bridge the democratic gap of CV care in underdeveloped and developing countries.^55^ Any perspective deployment of AI in cardiology remains, however, speculative due to many gaps that need to be filled.

### 5.1 Technical and regulatory gaps

**FIGURE 1:**
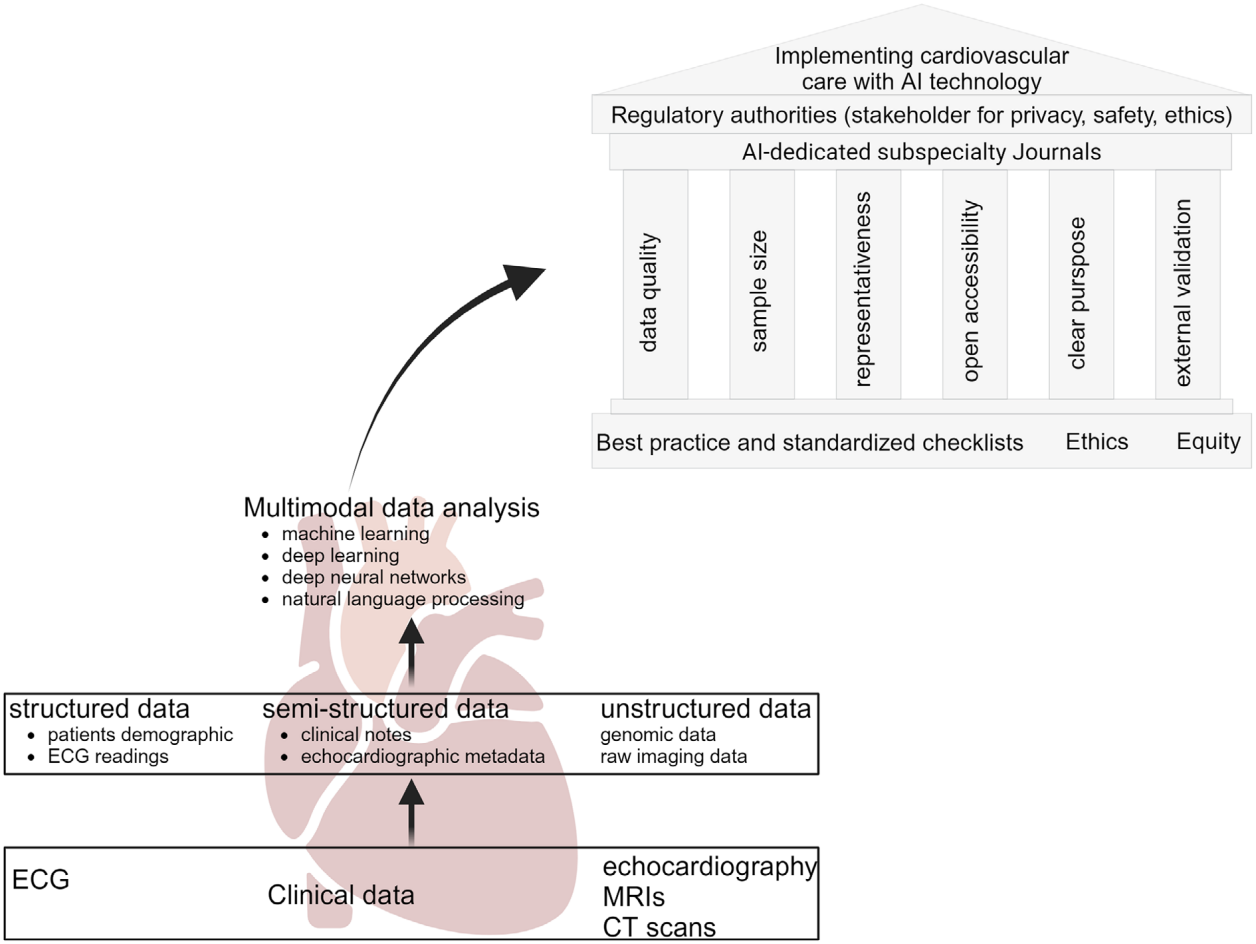
Present and future steps for implementing cardiovascular care with AI technology. Advances in cardiovascular technology enable large‐scale data collection from sources such as electrocardiography (ECG), clinical records and imaging modalities (e.g. echocardiograms, magnetic resonance imaging and computed tomography). While multimodal data analysis progresses with deeper exploration of machine learning algorithms, unmet needs persist, requiring standardized regulations from recognized regulatory authorities. Created with BioRender.com.

The ESC President Thomas F. Lüscher has recently provided a roadmap that ML must comply with as it moves into clinical practice. We strongly endorse such high‐quality standards, which emphasize data quality (i), size (ii), representativeness (iii) and open accessibility (iv). Further, these data must be used for a clear intended purpose (v) and integrated with externally validated algorithms (vi) that constantly learn as cohort characteristics change over time.^6^ While most AI‐based tools in CV research still do not meet these standards potentially harming patients,^56^ more than one hundred AI‐based prospective and randomized clinical trials are underway in the CV field (e.g. ECG‐AI, device‐embedded algorithms, multimodal wearable devices).^57^ Significant efforts are being made to develop dedicated guidelines following best practice and standardized checklists (e.g. CONSORT‐AI, SPIRIT‐AI, PRIME).^58^, ^59^, ^60^ Although the editorial boards of AI‐dedicated subspecialty journals, such as those launched by *The Lancet*, *New England Journal of Medicine* and *European Heart Journal*, may help to identify low‐standard or deceptive use of AI, the evolving landscape towards clinical and commercial purposes require governance and regulatory authorities to enforce specific interventions (Figure 1). The EU AI Act (approved in 2024, effective by 2026) limits the approval process to a pre‐marketing conformity assessment determined by risk categories,^61^ while the US act (late 2023) recognized the ‘model drift issue’ and calls for the development of assurance policy and structure dedicated to measuring both pre‐ and post‐marketing performance of AI models.^62^ Monitoring a model's performance is essential to recognizing any decline due to external changes, as advocated at the federal level. Since 2022, the Coalition for Health AI, a partnership of ex‐officio government members, has been fostering a nationwide conversation to help shape a network of assurance labs capable of fulfilling the requirements set forth by the US AI act.^63^

### 5.2 Ethical concerns

The deployment of AI in the CV field introduces ethical and equity concerns, which intersect with those mentioned above. As discussed elsewhere,^64^ the use of AI is susceptible to deceptive practices. The autonomy of cardiologists may be at risk with the implementation of AI in clinical care. For safe deployment, CV care providers need training in the use and limitations of the AI‐assisted workflows. It is conceivable to include AI courses already in the university and residency curricula to explicitly foster skills in data management and algorithm interpretation. This would also help clarify how physicians or providers are responsible for their use. Ethical concerns also extend to the equity of AI algorithm development and their responsible deployment. While anonymized and demographically sensitive algorithms should be developed—avoiding generic ‘debiasing’ methods and the use of uninterpretable algorithms—their application should emphasize complementing, rather than replacing, clinical judgement.^65^, ^66^, ^67^

## 6 CONCLUSION

The deployment of AI in CV medicine is likely unstoppable and has the potential to significantly improve patient care. Having already crossed this threshold, the question is no longer whether to adopt AI but how to effectively integrate it. This transformation is occurring across all areas of cardiology, and the advent of multimodality holds promise for streamlining and accelerating its integration into existing workflows.

Beyond reshaping predictive scores, AI‐powered clinical trials have the potential to deeply enhance evidence translation. Applications such as the screening of large electronic health records (EHRs), optimizing patient allocation and automating time‐consuming, error‐prone processes exemplifies how ML and NLP can optimize clinical trials. These technologies also drive innovation in adaptive clinical trials, data‐driven prediction enrichment and the emulation of traditional trial methods.

However, the ‘black box’ nature of AI undoubtedly poses challenges to clinicians, which must be addressed at the regulatory level. There is a growing emphasis on the need for models to balance performance across broad populations while maintaining individual specificity. The inherent variability of conditions across different demographics can significantly impact an algorithm's predictive accuracy at the individual level. Adaptive algorithms, often referred to as “locally validated,” are one approach to this issue. Furthermore, evidence in the literature supports recurrent assessments and recalibrations of models, tailored to both sex‐specific and regional population data, to enhance individualized care.

The frequent lack of clear anatomical and physiological explanations for AI‐derived models highlights the need for transparency, enabling clinicians to understand the foundation of AI‐driven decisions. Implementing transparency is essential to clarify liability by delineating the roles of the algorithm and human oversight. Mechanisms that enhance model explainability and ensure regular assessments of AI algorithms—such as post‐market surveillance with periodic revalidation and real‐time monitoring in clinical practice—could help address these liability concerns by ensuring models are continuously validated against real‐world data. Collaboration between AI developers, clinicians and regulatory authorities is crucial to establish standardized practices for managing liability in cases of AI‐related errors and to define a clearer framework for assigning responsibility.

## AUTHOR CONTRIBUTIONS

AS, DR and FC wrote the manuscript; LL and FM provided a critical revision of the paper.

## FUNDING INFORMATION

Work funded by #NEXTGENERATIONEU (NGEU), Ministry of University and Research (MUR), National Recovery and Resilience Plan (NRRP), project MNESYS (PE0000006) – A Multiscale integrated approach to the study of the nervous system in health and disease (DN. 1553 11.10.2022); recipient Federico Carbone and Fabrizio Montecucco. This research was also funded by a grant from the Rete Cardiologica of Italian Ministry of Health (RCR‐ 2022‐23682288) to Fabrizio Montecucco.

## CONFLICT OF INTEREST STATEMENT

Luca Liberale is co‐inventor on the international patent WO/2020/226993 filed in April 2020. The patent relates to the use of antibodies which specifically bind IL‐1α to reduce various sequelae of ischemia–reperfusion injury to the central nervous system. Luca Liberale reports speaker fees from Daiichi‐Sankyo outside the submitted work and has received funding from the Novartis Foundation for Medical‐biological Research (unrelated to this work). The other authors declare they have no conflict of interest.

## Data Availability

Not applicable.
